# Report of Mortality in Buffalo for the Month of Nov., 1856

**Published:** 1857-01

**Authors:** C. L. Dayton

**Affiliations:** Health Physician


ART. X.—*Report of Mortality in Buffalo for the month of Nov.*, 1856. By C. L. DAYTON, M. D., Health Physician.

| DISEASES. | No. | Males. | Females. |
|---|---|---|---|
| Accidens, | 2 | 2 | |
| Angina Maligna, | 1 | | 1 |
| Asphyxia Immersorum, | 2 | 2 | |
| Apoplexia, | 1 | 1 | |
| " Pulmonalis, | 1 | 1 | |
| " Cerebralis, | 1 | 1 | |
| Burn, | 2 | 1 | 1 |
| Bronchitis, | 1 | | 1 |
| " Chronica, | 1 | | 1 |
| Cynanche Trachealis, | 2 | | 2 |
| Cholera Infantum, | 1 | 1 | |
| Cancer, | 1 | | 1 |
| Catalepsy, | 1 | | 1 |
| Dentitio, | 1 | | 1 |
| Dyspepsia, | 1 | 1 | |
| Debilitas consequent on injuries on head, | 3 | 1 | 2 |
| " " consequent of injuries on head, | 1 | 1 | |
| Diarrhœa Chronica, | 1 | | 1 |
| Diptheritis, | 1 | 1 | |
| Epilepsy, | 2 | 1 | 1 |
| Enteritis, | 1 | | 1 |
| Eclampsia, | 11 | 8 | 3 |
| " Puerperarum, | 1 | | 1 |
| Fracture Cranii, | 1 | 1 | |
| Febris Typhus, | 1 | | 1 |
| " Typhoid, | 1 | | 1 |
| " Scarlatina Maligna, | 7 | 3 | 4 |
| " Scarlatina et Hydrops, | 13 | 4 | 9 |
| " Nervosa, | 1 | | 1 |
| " Intermittent, | 1 | | 1 |
| Hydrocephalus, | 2 | 1 | 1 |
| Hæmatemesis, | 1 | | 1 |
| Hyperæmia Pulmonalis, | 1 | | 1 |
| Hydrops, | 2 | 1 | 1 |

### REGISTER OF MORTALITY—Continued.

| DISEASES. | No. | Males. | Females. |
|---|---|---|---|
| Intestinal Rupture,, | 1 | | 1 |
| Intemperance and Exposure, | 2 | 1 | 1 |
| Ignotus, | 20 | 11 | 9 |
| Morbus Hepatis, | 2 | 1 | 1 |
| " Laryngee Chronica, | 1 | | 1 |
| Marasmus, | 10 | 7 | 3 |
| Narcosis, | 1 | | 1 |
| Peritonitis, | 5 | 2 | 3 |
| Parturitio, | 1 | | 1 |
| Paralysis, | 1 | 1 | |
| Pleuritis Chronica, | 1 | 1 | |
| Premature Birth, | 1 | | |
| Scrophula, | 2 | 1 | 1 |
| Still-born, | 7 | 2 | |
| Spina Bifida, | 1 | 1 | |
| Tuberculosis Pulmonalis, | 16 | 8 | 8 |
| " Laryngee, | 2 | 1 | 1 |
| Total, | 145 | | |

### SEXES.

| | |
|---|---|
| Males, | 69 |
| Females, | 70 |
| Sex not given, | 6 |
| Total, | 145 |

### AGES.

| | | | |
|---|---|---|---|
| Still-born, | 7 | Between 10 years and 20 years, | 7 |
| 1 day, | 0 | " 20 " " 30 " | 15 |
| Between 1 day and 30 days, | 10 | " 30 " " 40 " | 14 |
| " 1 month and 6 months, | 15 | " 40 " " 50 " | 8 |
| " 6 months and 12 " | 10 | " 50 " " 60 " | 8 |
| " 1 year " 3 years, | 27 | " 60 " " 70 " | 2 |
| " 3 " " 5 " | 11 | " 70 " " 80 " | 1 |
| " 5 " " 10 " | 5 | " 80 " " 90 " | 2 |
| | | " 90 " " 100 " | 0 |
| Total number under 10 years, | 85 | Total number over 10 years, | 57 |
| Ages not given, | 0 | Total, | 145 |

### NATIVITIES.

| | | | |
|---|---|---|---|
| American, (white, 49), colored, 1, | 50 | Prussian, | 0 |
| German, | 45 | French, | 2 |
| Irish, | 19 | Scotch, | 2 |
| English, | 3 | Holland, | 1 |
| Canadian, | 2 | Country not given, | 1 |
| Total, | | | 145 |

